# Performance of two interferon-gamma release assays for tuberculosis infection screening in Kawasaki children before immunosuppressive therapy

**DOI:** 10.3389/fped.2023.1162547

**Published:** 2023-05-18

**Authors:** Hao Chen, Huiwen Zheng, Lang Cui, Jing Xiao, Feina Li, Yonghong Wang, Yajie Guo, Yuying Chen, Yue Yuan, Chen Shen

**Affiliations:** ^1^ Laboratory of Respiratory Diseases, Beijing Key Laboratory of Pediatric Respiratory Infection Diseases, Beijing Pediatric Research Institute, Beijing Children’s Hospital, Capital Medical University, Key Laboratory of Major Diseases in Children, Ministry of Education, National Clinical Research Center for Respiratory Diseases, National Center for Children’s Health, Beijing, China; ^2^ Department of Cardiology, Beijing Children’s Hospital, Capital Medical University, National Center for Children’s Health, Beijing, China,

**Keywords:** interferon-gamma release assays, children, Kawasaki disease, tuberculosis, immunosuppressive therapy

## Abstract

**Objective:**

We aimed to compare QuantiFERON-TB Gold In-Tube (QFT-GIT) and X.DOT-TB for screening latent tuberculosis infection (LTBI) in kawasaki patients, and to identify the risk factors associated with indeterminate IGRA results.

**Methods:**

We conducted a retrospective study on children with KD, who were screened for mycobacterium tuberculosis (Mtb) infection by either ELISA-based QFT-GIT or ELISPOT-based X.DOT-TB tests, admitted in Department of Cardiology, Beijing Children's Hospital from July 2019 to April 2022.

**Results:**

A total of 1327 cases were included. Among them, 932 cases were tested by QFT-GIT and 395 cases by X.DOT-TB. The positive rate of children was 0.1% and 0.2%, and the indeterminate rate was 68.2% and 6.1% for QFT-GIT and X.DOT-TB, respectively. Patients with hypoproteinemia had a higher risk of indeterminate X.DOT-TB result. Female, critical ill, shock or hypoproteinemia presented statistically significant associations with an increased risk of indeterminate QFT-GIT result. High-dose of IVIG inhibited the release of IFN-*γ* by more than 90%, which might account for the high indeterminate incidence.

**Conclusion:**

It is recommended to perform X.DOT-TB rather than QFT-GIT to screen LTBI in patients with high level of the mitogen that can inhibit IFN-*γ* release. For KD children with positive IGRA results, it has a higher risk of activation TB infection when treated with immunosuppressive therapy in the future. Children with KD aged <5 years old had higher frequency of indeterminate IGRA results.

## Introduction

Kawasaki disease (KD), an acute systemic vasculitis of unknown etiology, mainly involved in the middle and arterioles in children under 5 years old (1, 2). Current standard treatment includes intravenous immunoglobulin (IVIG), alongside aspirin (3, 4). Other medications include glucocorticoids (e.g., high-dose pulsed intravenous methylprednisolone), tumour necrosis factor (TNF)-alpha inhibitors (e.g., infliximab), interleukin (IL)-1 inhibitors, (e.g., anakinra), or ciclosporin, especially for IVIG-resistant cases (5–8).

According to world health organization (WHO), there was approximately 1.2 million TB cases in children in 2021 (9). Children and young adolescents (aged below 15 years) represent about 11% of all people with tuberculosis (TB) globally. This means that 1.1 million children become ill with TB every year, almost half of them below five years of age (10). According to a previous report in Beijing, the average annual TB morbidity was 31.67/10,000 (11). Although mycobacterium tuberculosis (Mtb) infected individuals have a possibility of 5%–10% to develop active TB, immunosuppressive therapy by glucocorticoids, TNF-alpha inhibitors, and some others, have been proved to increase the risk of active TB disease in latent tuberculosis infection (LTBI) individuals. Though being preventable and curable, TB remains one serious threat to children's health (12–14). Young children, whose immune system is still at the stage of development, are much more likely to develop severe or disseminated TB following infection, and the situation could be more complicated if it occurs in combination with basic diseases. So it is necessary and highly recommended to screen Mtb infection in KD children before immunosuppressive therapy.

The interferon-γ release assays (IGRAs) detects the production of interferon-gamma (IFN-γ) by effector T cells stimulated by Mtb-specific antigens, and are reliable immune tests for identification of Mtb infection. At present, there are two main kinds of IGRAs with different experimental process and interpretation of the results. One measures the concentration of IFN-γ via an Enzyme-linked Immunosorbent Assay (ELISA) and another measures the frequencies of IFN-γ-secreting cells via Enzyme-linked Immunospot Assay (ELISPOT) (15). In our hospital, two commercial kits of IGRAs were used clinically. Among them, QuantiFERON-TB Gold In-Tube (QFT-GIT) assay (QIAGEN, Australia) is ELISA based, while X.DOT-TB (TB Healthcare, Foshan, China) is ELISPOT based (16). Patients were routinely screened in our hospital by either IGRAs before immunosuppressive therapy.

We aimed to compare QFT-GIT and X.DOT-TB for screening LTBI in kawasaki patients admitted in Department of Cardiology, Beijing Children's Hospital from July 2019 to April 2022, and to identify the risk factors associated with indeterminate IGRA results.

## Materials and methods

### Ethic statements

This study was approved by the Ethics Committee of Beijing Children's Hospital, Capital Medical University.

### Study population

We conducted a retrospective study on children aged ≤18 years with KD, who were screened for Mtb infection admitted and hospitalized in Department of Cardiology, Beijing Children's Hospital affiliated to Capital Medical University from July 2019 to April 2022.

### Patient categories

The diagnostic criteria for Kawasaki disease is according to Japanese Guidelines for Kawasaki Disease (Sixth revision) (17). Here are six of its latest key clinical features: (1) Fever; (2) Bilateral bulbar conjunctival injection; (3) Changes of lips and oral cavity: reddening of lips, strawberry tongue, diffuse injection of oral and pharyngeal mucosa; (4) Rash [including redness at the site of Bacille Calmette Guerin (BCG) inoculation]; (5) Changes of peripheral extremities: (Initial stage) reddening of palms and soles, edema. (Convalescent stage) periungual desquamation; (6) Non-supparative cervical lymphadenopathy.

Complete KD (Classic KD) subgroup: (1) Fulfill five or six of the above signs is diagnosed with complete KD; (2) Fulfill four of the above signs and coronary artery abnormality by echocardiography is diagnosed with complete KD.

Incomplete KD (Atypical KD) subgroup: KD is classified as incomplete (atypical) when the symptoms and signs of the child satisfy some but not all diagnostic criteria for complete KD (18). When the patients who fulfill three or four signs in the principle clinical features without coronary artery dilation but with some features from the list of “Other significant clinical features can be diagnosed as incomplete KD, if other diseases are ruled out; 2) Incomplete KD may also be considered in the presence of only one or two principal clinical features after excluding other diagnoses (17).”

Refractory KD (IVIG-resistant) subgroup: Refractory Kawasaki disease diagnosed when fever persists or recurs of any magnitude 24–36 h after completion of IVIG therapy (18, 19).

Age groups: According to the who 2022 Guidelines for the Management of tuberculosis in Children and Adolescents, the children were divided into infants (less than 1 year old), younger children (less than 5 years old), children (less than 10 years old), and younger adolescents (10–15 years old) (10).

### Interferon-gamma release assays

X.DOT-TB: X.DOT-TB is a commercial kit of ELISPOT-based IGRA. Results are reported as the number of IFN-γ-producing T cells (spot-forming cells). Peripheral blood mononuclear cells (PBMCs) obtained from each subject within 4 h of collection were seeded (2.5 × 10^6^ cell/ml) on a plate precoated with the antibody against IFN-γ. A nil control well, a positive control well with mitogen (phytohemagglutinin), and a TB antigen well (containing ESAT-6, CFP-10) were needed for each sample. Plates were incubated for 20–22 h at 37°C in 5% carbon dioxide. After incubation, wells were developed with a conjugate against the antibody used and an enzyme-substrate. Spot-forming cells (SFCs) were counted with an automated ELISpot reader (AID-ispot, Strassberg, Germany). An individual was considered positive for Mtb infection if the spot count in the TB antigen well exceeded a specific threshold relative to the negative control well. The results were interpreted in Table 1.

**Table 1:** Characteristics of IGRAs and result interpretations.

Variable	QFT-GIT	X.DOT-TB
**Format**	ELISA	ELISPOT
**Specimen**	Whole Blood (1 ml per tube, 3 tubes)	PBMCs (2.5 × 10^6^ per well, 4 wells)
**TB antigens**	ESAT6,CFP10,TB7.7	ESAT6,CFP10
**Posetive control**	Mitogen(Phytohemagglutinin)	Mitogen(Phytohemagglutinin)
**Output**	IU/ml	Spot forming units(SFU)
**Positive result**	*N* < 8.0 and TB-N ≥ 0.35 IU/ml, and >25% of nil	20 > *N* ≥ 11 and T-N ≥ N
**Indeterminate result**	*N* < 8.0 and M-N < 0.5 IU/ml; *N* > 8.0	*N* > 20 Spots in Nil; T-N < *N*(11 < *N* < = 20)

*N*, Spot count in nil control; TB-N, TB Antigen minus Nil (IU/ml); T-N, Spot count of test minus nil control; M-N, Mitogen(phytohemagglutinin) minus Nil (IU/ml).

QFT-GIT: According to the manufacturer's instructions, 1 ml of whole blood was collected into each of the three separate test tubes, including a nil control tube, a positive control tube with phytohemagglutinin, and a TB antigen tube (containing ESAT-6, CFP-10 and TB7.7), followed by incubated for 16–24 h at 37°C. Then the tubes were centrifuged and the supernatant were collected to assess the concentration of IFN-γ (IU/ml) via ELISA. The results were interpreted in Table 1.

### Determination of IgG concentrations in plasma

Concentrations of IgG were confirmed using the commercial kit of Tina-quant IgG Gen.2 from Beckman Coulter (USA) Co., Ltd, following the respective manufacturer's instructions. Each blood sample was divided into two parts, one with IVIG and the other with the same volume of saline, and then tested simultaneously. The experiment was divided into high and low dose groups according to the different concentrations of IVIG. The final concentration of IVIG was 15 mg/ml in the high dose group and 5 mg/ml in the low dose group [15.2 ± 1.3 mg/ml (mean ± SD) for high dose; 5.1 ± 1.0 mg/ml for low dose by ELISA determinant].

### Statistical analysis

Categorical variables were presented as percentages, while continuous variables were presented as means and standard deviations. *P*-values <0.05 were considered statistically significant. Multivariable models were built using “Enter” logistic regression procedures. Data analyses were conducted using SPSS version 23.0.

## Results

### Characteristics of enrolled patients

A total of 1,327 hospitalized children with KD, who were screened for Mtb infection by either of IGRAs at admission, were included in this study. Among them, 932 cases were tested by QFT-GIT and 395 cases were tested by X.DOT-TB, respectively. QFT-GIT group includes 591 males and 341 females, while X.DOT-TB group has 250 males and 145 females. Children aged 1 to 5 years (65.0%) and with classic KD (75.4%) were more common in our subjects. Details were presented in Table 2.

**Table 2:** Baseline characteristics of the study population.

	X.DOT-TB (*N* = 395)	QFT-GIT (*N* = 932)	Total (*N* = 1,327)
	*n* (%)	*n* (%)	*n* (%)
**Gender**			
M	250 (63.2)	591 (63.4)	841 (63.4)
F	145 (36.7)	341 (36.6)	486 (36.6)
**Age groups, y**			
0–1	65 (16.4)	162 (17.4)	227 (17.1)
1–5	260 (65.8)	602 (64.6)	862 (65.0)
5–10	65 (16.4)	158 (17.0)	223 (16.8)
10–15	5 (1.3)	10 (1.1)	15 (1.1)
**KD classification**			
Refractory	52 (13.1)	106 (11.4)	158 (11.9)
Classic	256 (64.8)	745 (79.9)	1,001 (75.4)
Atypical	87 (22.0)	85 (9.1)	172 (12.9)

### Overall results of IGRA assays

**Figure 1:**
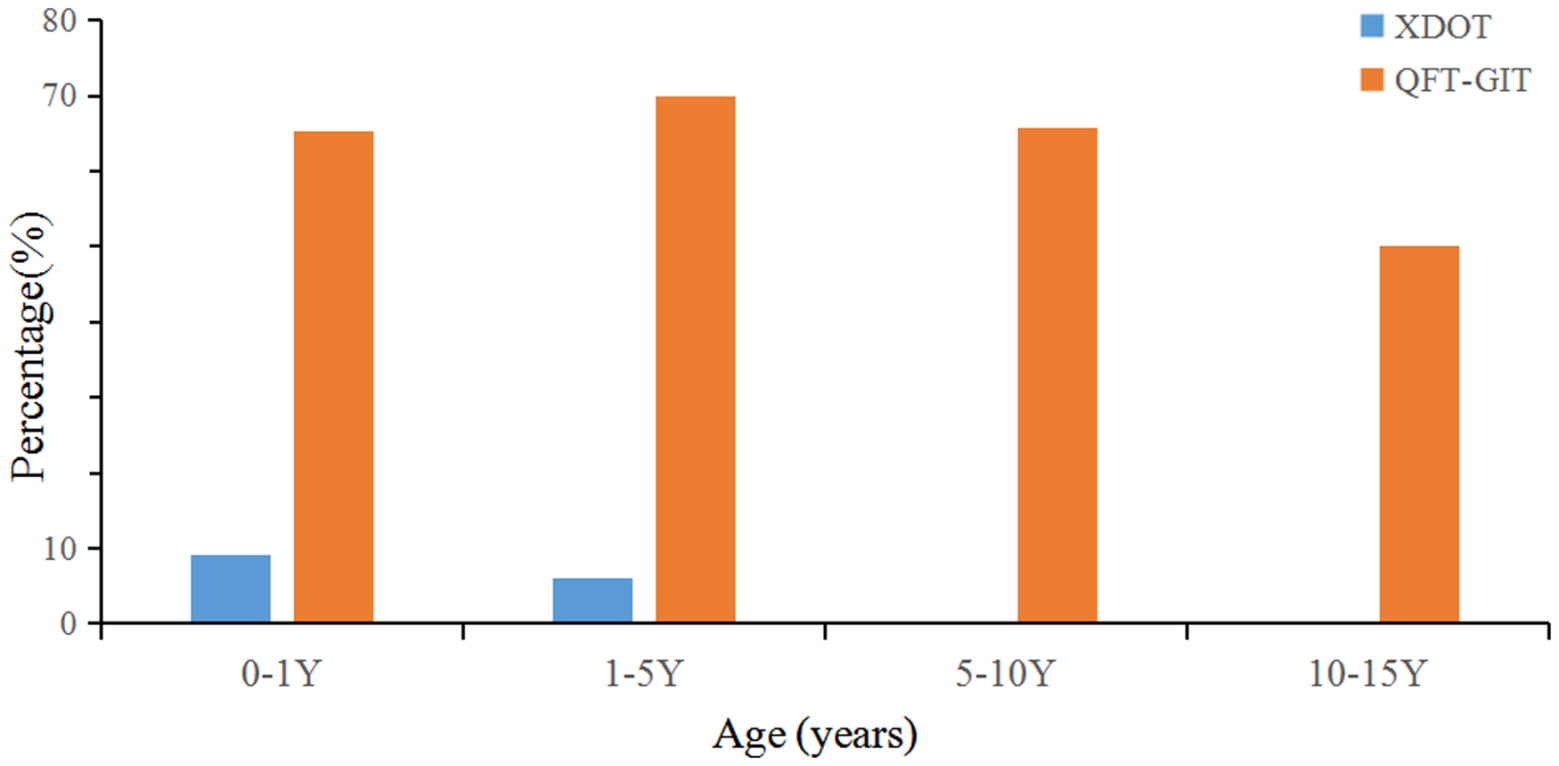
Percentages of indeterminate QFT-GIT and X.DOT-TB results by age groups.

The positive rate of Mtb infection for children with KD was 0.1% detected by QFT-GIT and 0.2% by X.DOT-TB respectively. And the indeterminate rate of QFT-GIT and X.DOT-TB was 68.2% and 6.1% respectively (Table 3). The proportion of QFT-GIT indeterminate results in children aged 1 to 5 years (69.9%) was greater than that in other age groups. And the indeterminate results of X.DOT-TB for children under 1 year (9.1%) was greater than that in other age groups. A comparison of X.DOT-TB vs. QFT-GIT indeterminate results by age groups can be found in Figure 1. The proportion of QFT-GIT indeterminate results was much higher than that of X.DOT-TB results across age groups (*P *< 0.001).

**Table 3:** The general results of X.DOT-TB and QFT-GIT assays.

Variable	X.DOT-TB				QFT-GIT				*P*_Indeterminate_ (X.DOT-TB vs. QFT-GIT)
	Total *n*	Determinate		Indeterminate *n* (%)	Total *n*	Determinate		Indeterminate *n* (%)	
		Positive, *n* (%)	Negative, *n* (%)			Positive *n* (%)	Negative, *n* (%)		
**Gender**									
F	250	0 (0.0)	232 (92.8)	18 (7.2)	591	1 (0.2)	200 (33.8)	390 (66.1)	<0.001
M	145	1 (0.6)	138 (95.9)	6 (4.1)	341	0 (0.0)	95 (27.8)	246 (72.1)	<0.001
**Age groups**									
0–<1	65	0 (0.0)	58 (90.8)	7 (9.1)	162	0 (0.0)	56 (34.6)	106((65.4)	<0.001
1–<5	260	1 (0.3)	242 (93.8)	17 (6.1)	602	1 (0.1)	180 (29.9)	421 (69.9)	<0.001
5–<10	65	0 (0.0)	65 (100)	0 (0.0)	158	0 (0.0)	54 (34.2)	104 (65.8)	<0.001
10–<15	5	0 (0.0)	5 (100)	0 (0.0)	10	0 (0.0)	5 (50)	5 (50)	0.053
**KD Classification**									
Refractory	52	0 (0.0)	51 (98.1)	1 (1.9)	106	1 (0.9)	20 (18.8)	85 (80.2)	<0.001
Classic	256	1 (0.3)	237 (92.6)	18 (7.0)	745	0 (0.0)	236 (31.7)	509 (68.3)	<0.001
Atypical	87	0 (0.0)	84 (96.5)	3 (3.4)	85	0 (0.0)	40 (47.1)	45 (52.9)	<0.001
Total	395	1 (0.2)	370 (93.7)	24(6.1)	932	1(0.1)	295(31.7)	636(68.2)	<0.001

### Risk factors for an indeterminate IGRA result

Univariable logistic regression analysis revealed that children with hypoproteinemia were more likely to have an indeterminate X.DOT-TB result (16. 0%) than that without hypoproteinemia (4. 8%) (*P *= 0. 02). Multivariate logistic modelling analysis showed that children with hypoproteinemia had a higher risk of indeterminate X.DOT-TB result [adjusted odds ratio (aOR) 8. 90, 95% CI 3. 03–26. 11] (Table 4).

**Table 4:** Multivariate analysis of risk factors associated with indeterminate X.DOT-TB results in children with KD.

Characteristic	Indeterminate (*n* = 22)	Determinate (*n* = 373)	Univariable analysis		Multivariate analysis	
			Crude OR (95% CI)	*P-*value	Adjusted OR (95% CI)	*P-*value
**Age groups**						
0–<1	6 (9.1)	59 (90.9)	1.10 (1.02–1.19)	0.48	–	1.00
1–<5	16 (6.1)	244 (93.9)	1.07 (1.03–1.10)	0.57	–	1.00
5–<10	0	65 (100.0)	^a^	^a^	^a^	^a^
10–<15	0	5 (100.0)	Reference	–	Reference	–
**Gender**						
F	6 (4.0)	139 (96.0)	0.63 (0.24–1.65)	0.35	0.79 (0.40–1.55)	0.49
M	16 (6.4)	234 (93.6)	Reference	–	Reference	–
**KD Subtypes**						
Refractory	1 (1.9)	51 (98.1)	0.55 (0.06–5.42)	0.60	0.06 (0.01–0.62)	0.02
Classic	18 (7.0)	238 (93.0)	2.12 (0.61–7.37)	0.23	0.51 (0.23–1.16)	0.11
Atypical	3 (3.4)	84 (96.5)	Reference	–	Reference	–
**Critically ill**						
Y	15 (6.7)	209 (93.3)	1.68 (0.67–4.22)	0.26	1.57 (0.23–1.16)	0.19
N	7 (4.0)	164 (96.0)	Reference	–	Reference	–
**In shock**						
Y	1 (5.5)	17 (94.5)	1.00 (0.13–7.86)	1.00	0.35 (0.06–2.01)	0.24
N	21 (5.5)	356 (94.5)	Reference	–	Reference	–
**Hypoproteinemia**						
Y	4 (16.0)	21 (84.0)	3.73 (1.56–12.00)	0.02	8.90 (3.03–26.11)	＜0.01
N	18 (4.8)	352 (95.2)	Reference	–	Reference	–
**Coronary artery dilatation**						
Y	6 (7.8)	70 (92.2)	1.62 (0.61–4.30)	0.33	1.51 (0.74–3.09)	0.26
N	16 (5.0)	303 (95.0)	Reference	–	Reference	–
**With conduction block**						
Y	1 (3.7)	26 (96.3)	0.64 (0.08–4.91)	0.66	0.55 (0.12–2.46)	0.43
N	21(5.7)	347(94.3)	Reference	–	Reference	–

^a^ No statistics are computed as result is a constant.

For QFT-GIT, using children with atypical KD as a control group, univariable logistic regression analysis revealed that children with refractory KD and classic KD compared with atypical KD, were more likely to have an indeterminate result (80. 2%, 68. 3%, and 52. 9%, respectively). Besides, children with shock or hypoproteinemia had significantly higher odds of having an indeterminate result than that without shock or hypoproteinemia, respectively. Among the risk factors analyzed through logistic regression, female, children with critical ill, shock or hypoproteinemia presented a statistically significant association with an increased risk of obtaining an indeterminate QFT-GIT result (Table 5).

**Table 5:** Multivariate analysis of risk factors associated with an indeterminate QFT-GIT result in children with KD.

Characteristic	Indeterminate (*n* = 636)	Determinate (*n* = 296)	Univariable analysis		Multivariate analysis	
			Crude OR (95% CI)	*P-*value	Adjusted OR (95% CI)	*P-*value
**Age groups**						
0–<1	106 (65.4)	56 (34.6)	1.89 (0.53–6.82)	0.32	1.81 (0.71–4.63)	0.22
1–<5	421 (69.9)	181 (30.1)	2.33 (0.67–8.13)	0.17	2.20 (0.88–5.50)	0.09
5–<10	104 (65.8)	54 (34.2)	1.93 (0.53–6.94)	0.31	1.67 (0.65–4.27)	0.29
10–<15	5 (50.0)	5 (50.0)	Reference	–	Reference	–
**Gender**						
F	246 (72.1)	95 (38.6)	1.34 (1.00–1.79)	0.05	1.42 (1.14–1.76)	<0.01
M	390 (66.0)	201 (51.5)	Reference	–	Reference	–
**KD Subtypes**						
Refractory	85 (80.2)	21 (19.8)	3.60 (1.90–6.82)	<0.01	2.14 (0.84–5.48)	0.11
Classic	509 (68.3)	236 (31.7)	1.92 (1.22–3.02)	<0.01	1.17 (0.53–2.58)	0.69
Atypical	45 (52.9)	40 (47.1)	Reference	–	Reference	–
**Critically ill**						
Y	411 (64.6)	177 (27.8)	1.23 (0.93–1.63)	0.16	1.26 (1.02–1.55)	0.03
N	225 (76.0)	119 (40.2)	Reference	–	Reference	–
**In shock**						
Y	18 (94.7)	1 (5.3)	8.59 (1.14–64.67)	0.01	5.88 (1.36–25.41)	0.02
N	618 (67.7)	295 (32.3)	Reference	–	Reference	–
**Hypoproteinemia**						
Y	47 (90.4)	5 (10.6)	4.64 (1.83–11.80)	<0.01	4.88 (2.42–9.82)	<0.01
N	589 (66.9)	291 (49.4)	Reference	–	Reference	–
**Coronary artery dilatation**						
Y	132 (68.8)	60 (31.3)	1.03 (0.73–1.45)	0.87	0.93 (0.72–1.20)	0.56
N	504 (68.1)	236 (31.9)	Reference	–	Reference	–
**With conduction block**						
Y	45 (71.4)	18 (28.6)	1.18 (0.67–2.07)	0.57	1.09 (0.73–1.63)	0.67
N	591(68.0)	278(32.0)	Reference	–	Reference	–

KD classification does not present a statistically significant *P-*value in multivariate logistic modelling to be associated with the incidence of an indeterminate QFT-GIT result, which may because the disease classification could be somewhat symptoms dependent, but an eighty percent indeterminate rate in refractory KD subgroups is really high.

### IVIG concentration in blood and the release of IFN-γ

**Figure 2:**
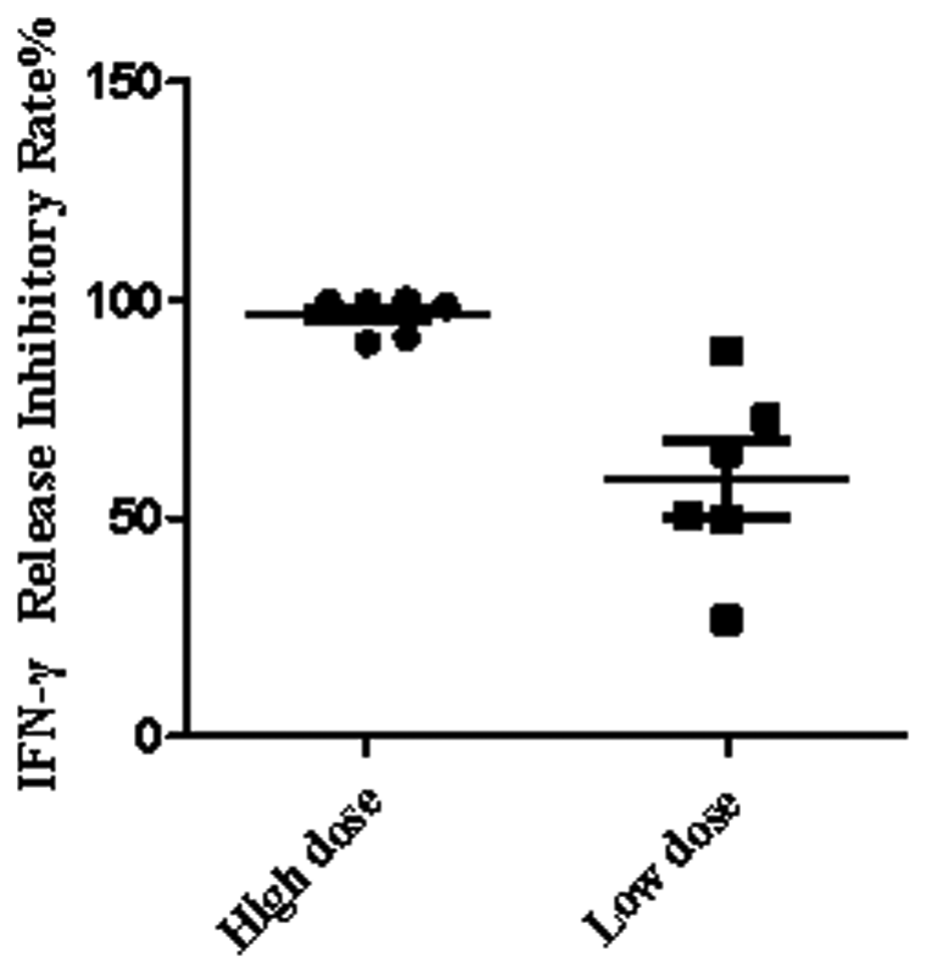
IFN-γ release inhibitory rate by IVIG.

IVIG is one of the first-line KD therapeutic drugs. It could be given during emergency treatment before hospitalization or in primary hospitals before referral to our hospital. Cases being less response 24–36 h after completion IVIG therapy would be diagnosed as Refractory KD. KD patients, especially those with refractory KD, presented a high indeterminate rate being less responsive to mitogen stimulation in blood by QFT-GIT, but not X.DOT-TB. We then measured the inhibitory effect of IVIG on Mitogen induced IFN-γ release. Compared to group without IVIG, the group added with high-dose IVIG inhibited IFN-γ secretion by more than 90% on average, but only about 50% in the group added with low-dose IVIG (Figure 2).

## Discussion

Screening for Mtb infection before immune-modulatory treatment is required for many diseases in hospital. A latent Mtb infection without symptoms could progress into active TB disease when the immune system being suppressed. ELISA-based QFT-GIT and ELISPOT-based X.DOT-TB are two types of IGRAs used in our hospital for distinguishing Mtb infected children. QFT-GIT seems to be more convenient and easier to perform than X.DOT-TB, as it uses whole blood rather than mononuclear cells for T cell stimulating (20). The positive rate of children detected by QFT-GIT and X.DOT-TB was not high according to our study, However, in this study, we found that the proportion of indeterminate IGRA results in KD by QFT-GIT was more than ten times higher than that by X.DOT-TB. We noticed all the indeterminate cases by QFT-GIT in this study were because of their response-less to mitogen treatment. QFT-GIT stimulates T cells in the whole blood, while X.DOT-TB stimulates T lymphocytes in extracted PBMCs by maintaining them in culture medium. Thus, increased indeterminate IGRA results by QFT-GIT means there could be certain components in the blood of KD patients that inhibit the stimulated release of IFN-γ. As IVIG is one of standard therapeutic drugs and most of the children especially refractory KD patients received IVIG therapy, we hypothesized that IVIG might influence IFN-γ release directly.

By adding IVIG to whole blood *in vitro*, we found that the presence of IVIG could directly inhibit the release of IFN-γ by mitogen stimulated T cells in the whole blood, and the effect is somewhat dose dependent. IFN-γ secretion were half reduced with low concentrations of IVIG, and sharply reduced with high-IVIG concentrations. The main principle of QFT-GIT assay is to detect the release level of IFN-γ in blood, whereas X.DOT-TB detects the mononuclear cells without plasma, which is not affected by increased IVIG content in whole blood. Thus, X.DOT-TB could be more suitable for children under IVIG treatment. As IVIG has been widely used in perinatal and neonatal periods in recent years (21, 22), attention should also be paid to children with similar situations. We suggest that a replacement of cell culture median for plasma might be helpful to improve the performance of ELISA-based IGRA method in children under IVIG treatment.

Due to immature immune function and limited compensatory ability, most children become seriously ill, develop rapidly and are prone to complications. In children with shock or hypoproteinemia, the indeterminate rate of QFT-GIT is more than 90%, so QFT-GIT is not recommended for Mtb infection screening when these conditions occur. In the case of hypoproteinemia, the indeterminate rate of X.DOT-TB was also elevated, reaching 16%. In this case, symptoms should be prioritized, and TB screening should be performed again when the signs are stable. The test results will be more accurate. Hypoproteinemia is associated with a higher risk for an indeterminate IGRA result by X.DOT-TB, while children with critical ill, shock or hypoproteinemia presented a statistically significant association with an increased risk of obtaining an indeterminate IGRA result by QFT-GIT. Albumin is a well-known negative acute phase reactant because its level decreases with inflammation, in which various relevant cytokines, such as IL-1, IL-6, and TNF-α, suppress the synthesis of albumin (23). Since our study did not test children with hypoalbuminemia, it only extrapolated from case statistics. Therefore, further research is needed to elucidate the mechanisms underlying the effects of low albumin levels on IGRA testing (24).

## Conclusions

It is recommended to perform X.DOT-TB rather than QFT-GIT to screen LTBI in patients with high level of the mitogen that can inhibit IFN-γ release. For KD children with positive IGRA results, it has a higher risk of activation TB infection when treated with immunosuppressive therapy in the future. Children with KD aged <5 years old had higher frequency of indeterminate IGRA results.

## Data Availability

The original contributions presented in the study are included in the article, further inquiries can be directed to the corresponding author.
